# Hand-held cell phone use while driving legislation and observed driver behavior among population sub-groups in the United States

**DOI:** 10.1186/s12889-017-4373-x

**Published:** 2017-05-12

**Authors:** Toni M. Rudisill, Motao Zhu

**Affiliations:** 1 Injury Control Research Center, West Virginia University, PO BOX 9151, Morgantown, West Virginia 26506 USA; 2 The Center for Injury Research and Policy, The Research Institute at Nationwide Children’s Hospital, 700 Children’s Drive, RB3-WB5217, Columbus, OH 43205 USA; 3 Department of Pediatrics, College of Medicine, Ohio State University, Columbus, OH 43210 USA

**Keywords:** Driving, Legislation, Cell phone, Epidemiology

## Abstract

**Background:**

Cell phone use behaviors are known to vary across demographic sub-groups and geographic locations. This study examined whether universal hand-held calling while driving bans were associated with lower road-side observed hand-held cell phone conversations across drivers of different ages (16–24, 25–59, ≥60 years), sexes, races (White, African American, or other), ruralities (suburban, rural, or urban), and regions (Northeast, Midwest, South, and West).

**Methods:**

Data from the 2008–2013 National Occupant Protection Use Survey were merged with states’ cell phone use while driving legislation. The exposure was presence of a universal hand-held cell phone ban at time of observation. Logistic regression was used to assess the odds of drivers having a hand-held cell phone conversation. Sub-groups differences were assessed using models with interaction terms.

**Results:**

When universal hand-held cell phone bans were effective, hand-held cell phone conversations were lower across all driver demographic sub-groups and regions. Sub-group differences existed among the sexes (*p*-value, <0.0001) and regions (*p*-value, 0.0003). Compared to states without universal hand-held cell phone bans, the adjusted odds ratio (aOR) of a driver hand-held phone conversation was 0.34 [95% confidence interval (CI): 0.28, 0.41] for females versus 0.47 (CI 0.40, 0.55) for males and 0.31 (CI 0.25, 0.38) for drivers in Western states compared to 0.47 (CI 0.30, 0.72) in the Northeast and 0.50 (CI 0.38, 0.66) in the South.

**Conclusions:**

The presence of universal hand-held cell phone bans were associated lower hand-held cell phone conversations across all driver sub-groups and regions. Hand-held phone conversations were particularly lower among female drivers and those from Western states when these bans were in effect. Public health interventions concerning hand-held cell phone use while driving could reasonably target all drivers.

## Background

In 2015, the global revenue from cell phone sales was in excess of $400 billion United States’ (U.S.) dollars [1]. More than 90% of U.S. residents possess a cell phone subscription [2] and over 60% of these subscribers owns a smartphone [3], which enables users unfettered access to information and communicative abilities such as calling, texting, emailing, and video chat. A 2015 national survey of cell phone owners revealed that nearly 50% of respondents said they could not live without this technology [2]. Therefore, there is little controversy that many Americans are enamored with their mobile devices.

While cell phones permit users with a constant connection to their environment and social circles, they also can serve as a constant distraction. National surveys suggest that many do not refrain from using this technology even in situations that may be hazardous, such as driving [4, 5]. There is a substantial body of literature that is comprised of experimental, epidemiologic, and naturalistic studies, which show cell phone use while driving (CPWD) negatively affects driving ability as it likely interferes with a driver’s visual, manual, and cognitive function [6–9]. Because the behavior is prevalent [10, 11] and has been increasing [11, 12], CPWD presents an enormous challenge for traffic safety and public health. Some have suggested that this may be due to the fact that perpetual cell phone use is a social norm [13].

As part of the federal government’s strategic plan to end CPWD, the U.S. Department of Transportation has actively encouraged states to pass CPWD legislation [14]. Consequently, states have ratified a myriad of CPWD laws consisting of bans on hand-held CPWD, texting while driving, and any cell phone use by young drivers (i.e. young driver all cell phone bans). As of October 2016, 14 states have enacted hand-held CPWD bans applicable to all drivers, 46 states have passed texting while driving bans for all drivers, and 37 states ban any type of cell phone use for young or inexperienced drivers (i.e. those with learner’s permit or intermediate licenses) [15].

Various studies have investigated the effectiveness of CPWD laws on reducing collision claims, fatal/injurious crashes, hospitalizations, road-side observed and self-reported CPWD behavior [9]. Because this study focuses on road-side observed driver behavior, only these types of studies shall be reviewed. To the authors’ knowledge, seven studies have scrutinized the effectiveness of CPWD bans on road-side observed driver behavior [16–22]. Two of these studies investigated the short and long term effects of a young driver all cell phone ban on phone usage among teenage drivers in North Carolina [17, 18]. In the first study, teenage drivers were observed one to 2 months before and then 5 months after the law passed [17]. The second study assessed the longer-term effects of these laws by observing teenage drivers at the same high schools originally sampled, but 2 years after the law’s implementation [18]. The first study found that the law had minimal short-term effects on driver phone usage [17]. In the second study, hand-held cell phone conversations occurred less overall, but the authors’ attributed this to the fact that texting was likely replacing talking on hand-held cell phones [18]. In regards to driver sex, these authors noted that female teen drivers were >60% more likely to talk on phones than males both before and after the law was passed [17, 18]. As for hand-held bans and road-side observed driver phone usage, five studies have explored this relationship [16, 19–22]. Four studies conducted by the Insurance Institute for Highway Safety investigated the short and long term effects of driver hand-held CPWD bans in Washington, DC and several New York and Connecticut communities [19–22]. In New York, hand-held CPWD initially decreased after the ban was enacted and then rose to pre-law levels 16 months later; the findings also showed that CPWD decreased among both sexes and across various age groups of drivers (i.e. <25, 25–59, and 60 year old drivers) [19, 20]. In Washington, DC, driver hand-held CPWD was greatly reduced both immediately after and for a year after the ban was ratified [21, 22]. In Connecticut, driver hand-held phone use was greatly reduced both immediately after and for almost 3.5 years after the passage of the law [22]. Only one study investigated road-side observed hand-held CPWD in a nationally representative sample of drivers between 2004 and 2010 using a difference-in-difference approach [16]. It appeared that hand-held bans were associated with decreased road-side observed hand-held CPWD; this relationship was consistent among drivers aged 16–24 and ≥25 years [16].

Collectively, these studies suggest that hand-held CPWD bans likely reduce road-side observed cell phone use among drivers. However, there are still extant gaps in the literature. It is not completely clear whether these cell phone laws are associated with reduced cell phone use behavior across different sub-groups of drivers, such as different ages, sexes, races/ethnicities, or even by location, such as rurality or region. Most of these studies did not formally test for sub-group differences. National surveys show that self-reported cell phone use varies by activity (i.e. if the person is calling, texting, emailing, viewing internet/apps, etc.) and also by age, sex, race/ethnicity, and rurality [23]. For example, texting behaviors do not differ by sex, but varies inversely with age, is less common in rural areas, and is more common among African Americans and Latinos compared to White Non-Hispanics [23]. National self-reported surveys of drivers reveal female drivers tend to text and have hand-held cell phone conversations less than males, and that both texting and hand-held phone conversations typically decrease as driver age increases [24]. Contrarily, road-side observed surveys of drivers from 2005 to 2014 show that females have consistently used hand-held phones to converse while driving more than males [10]. Research concerning other traffic safety laws has also shown that laws are not always equally obeyed amongst population sub-groups. It is well-established in the literature that younger drivers and males typically engage in riskier driving behaviors and receive more traffic citations, particularly when it comes to seat belt use, speeding, or driving too fast for conditions compared to females or middle-aged drivers [25, 26]. Race and ethnicity are often varied in fatal crashes concerning alcohol, seat belt non-use, and speeding [27] or with reciving traffic citations [28]. Previous studies have also shown that rural drivers tend to speed, not wear safety belts, and run stop signs/lights more than urban drivers [29, 30].

Because of these reported differences, the objective of this study was to investigate the association between hand-held CPWD laws and road-side observed hand-held cell phone conversations across driver sub-groups and regions. Because texting bans and young driver all cell phone bans may also influence driver behavior, this study controlled for these other bans. Given the existing research, it was hypothesized that universal hand-held bans may be associated with lower cell phone use in some driver sub-groups and regions. Determining this information is important for the development and tailoring of future public health interventions and/or public safety campaigns regarding hand-held cell phone use while driving.

## Methods

### Data sources

**Fig. 1:**
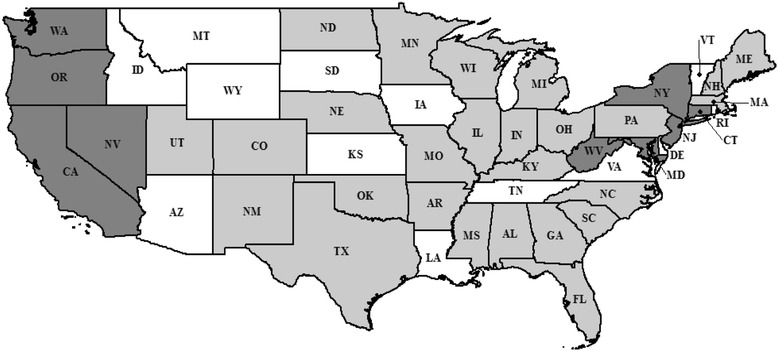
Map of states included in 2008–2013 NOPUS survey. Whilst the survey is nationally representative, not all states were sampled. States shaded in white were not sampled; Alaska and Hawaii were also not sampled (not shown). States in any shade of *grey* were sampled. States shaded in *dark grey* had a universal hand-held cell phone use while driving ban implemented before or during the survey period. Illinois and New Hampshire had universal hand-held bans, which became effective after the study period (January 2014 and July 2015, respectively)

The main data source for this analysis was the 2008–2013 National Occupant Protection Use Survey (NOPUS). Conducted by the National Highway Traffic Safety Administration (NHTSA), NOPUS is the only nationally representative road-side observed survey, which is conducted annually to assess driver and passenger safety behaviors including seat belt/child restraint use, motorcycle helmet use, and electronic device use; while data pertaining to driver cell phone use has been collected since 2000, the survey has undergone several methodological changes [31]. The survey involves a two-stage sampling design with stratified probability proportional to size; the number of primary sampling units and randomly selected observations sites vary each year [31]. Trained observers are dispatched to these sites, which are located at controlled intersections (i.e. at stop lights or stop signs) [31]. These observers assess the occupants of passenger vehicles during day light hours in June of each year [31]. In addition to the safety behaviors mentioned, occupants’ sex, age, and race are collected along with information such as the location of the intersection (i.e. rural, suburban, urban), traffic flow (heavy, light), etc. While the survey is nationally representative, not all states are sampled. A figure of the states sampled in the 2008–2013 NOPUS and those with universal hand-held bans are presented in Fig. 1; a list of each states’ dates when these universal hand-held bans became effective are included in the Appendix. This survey is described in more detail elsewhere [31]. The 2008–2013 NOPUS data were chosen for analysis because the survey methodologies were similar these years and many states passed cell phone legislation during this time. These data were made available and permitted for use upon request from the NHTSA’s Office of Behavioral Research.

In addition to the NOPUS data, a dataset of each states’ cell phone use while driving laws that were in effect from January 1, 2008 to December 31, 2013 was expended. This dataset was constructed by the study authors from several sources, including the Insurance Institute for Highway Safety and the Governor’s Highway Safety Association [15, 32, 33]. Each state law that was purported to exist was researched and retrieved from each state’s legislative archives, which are publically available on each individual states’ legislature websites. For accuracy purposes, two individuals verified and coded these laws for variables such as enactment dates, how the law was enforced, the ages of drivers the law affected, etc. In this analysis, the term ‘universal’ implies that the law applies to all licensed drivers.

To calculate cell phone coverage in states each year (i.e. number of cell phone subscriptions per 100 residents), the number of cell phone subscriptions per state per year were obtained from the Federal Communications Commissions’ Local Telephone Competition reports [34]. Population estimates per state by year were obtained from the U.S. Census Bureau [35]. These data are all publically available and can be obtained from the Federal Communication Commission and U.S. Census Bureau’s webpages.

### Study population and variables

Hand-held CPWD bans mainly do not permit drivers to make hand-held phone calls and converse on hand-held devices. The primary dependent variable of this analysis, which was dichotomous, was whether or not the observed driver was engaged in a hand-held cell phone conversation while driving. In NOPUS, roadside observers categorize drivers’ CPWD into 4 categories after ~10 s of observation: 1) driver was not using a cell phone, 2) driver was holding a hand-held cell phone to their ear and conversing, 3) driver was manipulating a hand-held device, or 4) driver was using a hands-free device [31]. Drivers holding hand-held cell phones to their ear and conversing were considered to be engaged in a hand-held cell phone conversation. Drivers who were not using hand-held cell phones or who were using hands free devices were considered to not be engaged in a hand-held cell phone conversation. Drivers manipulating hand-held devices were not included in order to minimize bias; it is virtually impossible from a road-side observation site to determine if a driver was text messaging, using the internet, etc. versus dialing a phone number or terminating a cell phone call. Also, hand-held devices in NOPUS can include electronic devices which may not always be a cell phone [31]. Throughout this manuscript, the term ‘hand-held cell phone use’ implies a driver was having a hand-held cell phone conversation.

The primary independent variable was dichotomized as to whether or not the state where the driver was observed had a universal hand-held cell phone ban in effect at time of observation. Other predictor variables, which were noted by roadside observers, included drivers’ age (<25, 25–60, ≥70 years of age), sex (male or female), race (White, African American, or other), location of observation (urban, suburban, or rural area), whether driver was wearing a seat belt (yes or no), and vehicle type (passenger car, pickup truck, van/sport utility vehicle). These categories, especially driver age, are somewhat broad because observers must categorize vehicle occupants from the roadside; broad categories minimize misclassification. The state where the observation took place was also categorized into geographic location (i.e. whether the state was in the Northeast, Midwest, West, or South); this was based on the U.S. Census region classification. The number of cell phone subscriptions per 100 residents was calculated by taking the number of subscriptions in each state divided by the states’ population and multiplied by 100 for each year. Year of driver observation was also included. Because other CPWD laws, such as universal texting bans or young driver all cell phone bans, may confound the relationship between hand-held CPWD and the presence of a hand-held ban, these laws were also separately taken into account. These laws were both dichotomized into whether or not a state had these bans at time of driver observation. Two states, Mississippi and Missouri, had texting bans during this time period that were not universal. These bans applied only to drivers under 21 years of age. These bans were also factored into the analysis as a dichotomous variable (i.e. present or absent at time of driver observation) called ‘non-universal texting laws’ as their presence may also have confounded the relationship between hand-held CPWD and a hand-held cell phone ban.

### Statistical analyses

The number of replicate weights differed for each year of the NOPUS data. In order to combine the 2008–2013 NOPUS data for analysis, pseudo strata and clusters were first created from the primary sampling units; the Taylor series approximation method was used to compute standard errors [36]. In order to determine whether hand-held cell phone bans were associated with hand-held cell phone use while driving, both crude and adjusted odds ratios were generated via logistic regression, which also accounted for the complex survey design of NOPUS (i.e. clusters and strata). Separate models were run for age, sex, race, location, and region. Models, which contained an interaction term, were run to assess for differences amongst the sub-groups and hand-held ban. Adjusted models accounted for survey year, sex, race, rurality, seatbelt use, vehicle type, presence of universal texting ban, presence of young driver all cell phone ban, presence of non-universal texting laws, and the number of cell phone subscriptions per 100 residents. All analyses were run in SAS version 9.4 with α = 0.05. The map of states, which shows the states whom were sampled in the 2008–2013 NOPUS, was created using ArcMap version 10.3.

## Results

Of the 263,673 drivers included in this analysis, 5.1% (*n* = 13,564) were talking on a hand-held phone at time of observation (Table 1). The majority of drivers were judged to be between 25 and 69 years of age (81.8%), male (58.6%), and of White race (81.4%). Most drivers were observed in suburban locations (57.1%) and were wearing safety belts (85.4%). Drivers having hand-held phone conversations tended to be younger, female, African American, and from Southern states compared to those not engaging in cell phone conversations. Over 72% of the drivers were from states where a hand-held ban was not present at time of observation. A list of states and their hand-held legislations’ effective dates are listed in Appendix, Table 6. Nearly half of all observed drivers were from states where universal texting bans and young driver bans were instituted at time of observation (50.4% and 55.2%, respectively).

**Table 1:** Characteristics of the roadside-observed drivers by hand-held phone status in 2008–2013 National Occupant Protection Use Survey^a,b^

	Driver not holding phone to ear or driver was using a head set (*N* = 250,109)		Driver holding phone to ears (*N* = 13,564)		Total (*N* = 263,673)	
Characteristic	N	(%)^b^	N	(%)	N	(%)
Age (years)						
16–24	29,799	(11.7)	2289	(16.5)	32,088	(12.0)
25–69	204,214	(81.8)	11,088	(82.1)	215,302	(81.8)
> 70	16,096	(6.5)	187	(1.4)	16,283	(6.2)
Sex						
Male	146,201	(59.2)	6383	(47.6)	152,584	(58.6)
Female	103,908	(40.8)	7181	(52.4)	111,089	(41.4)
Race						
White	202,036	(81.5)	10,777	(80.5)	212,813	(81.4)
Black	22,361	(8.3)	1673	(11.4)	24,034	(8.4)
Other	25,712	(10.3)	1114	(8.1)	26,826	(10.2)
Location						
Urban	42,447	(16.7)	2443	(17.6)	44,890	(16.8)
Suburban	148,174	(57.0)	8205	(59.9)	156,379	(57.1)
Rural	59,488	(26.3)	2916	(22.5)	62,404	(26.1)
Geographic region						
Northeast	63,736	(21.3)	2873	(16.6)	66,609	(21.1)
Midwest	57,188	(23.1)	3165	(22.5)	60,353	(23.1)
South	66,420	(28.5)	4708	(37.1)	71,128	(28.9)
West	62,765	(27.0)	2818	(23.8)	65,583	(26.9)
Seatbelt use						
Yes	214,358	(85.6)	11,059	(81.6)	225,417	(85.4)
No	35,751	(14.4)	2505	(18.4)	38,256	(14.6)
Vehicle type						
Passenger car	127,209	(49.6)	6405	(45.5)	133,614	(49.4)
Pick-up truck	41,094	(17.0)	2237	(17.3)	43,331	(17.0)
Van & SUV	81,806	(33.4)	4922	(37.2)	86,728	(33.6)
Cell phone subscriptions per 100 residents^c^	89		88		89	
Hand-held phone ban						
Yes	77,546	(28.3)	2132	(14.5)	79,678	(27.6)
No	172,563	(71.7)	11,432	(85.5)	183,995	(72.4)
Universal texting ban						
Yes	124,598	(50.8)	5310	(43.4)	129,908	(50.4)
No	125,511	(49.2)	8254	(56.6)	133,765	(49.6)
Young driver all cell phone ban						
Yes	130,264	(55.4)	6117	(51.0)	136,381	(55.2)
No	119,845	(44.6)	7447	(49.0)	127,292	(44.8)
Other texting bans						
Yes	3281	(1.7)	225	(1.5)	3506	(1.7)
No	246,828	(98.3)	13,339	(98.5)	260,167	(98.3)

^a^percentages may not add up to 100% due to rounding

^b^percentage is based on the weighted frequency

^c^the average number of cell phone subscribers per 100 residents

While drivers aged 16–24 years talked on hand-held devices more than other age groups regardless of hand-held ban existence, hand-held CPWD bans were associated with lower hand-held phone conversations across all age groups (Table 2).

**Table 2:** The association between driver hand-held cell phone conversations and state legislation stratified by age group

Characteristic	Total N^a^	Percent of drivers holding phone to ear^b^	Crude Odds Ratio (95% Confidence Limit)^c^		Adjusted Odds Ratio (95% Confidence Limit)^c^		*P*-value^d^
Hand-held phone ban in 16–24 year old drivers							0.7011
No	21,699	8.6	1.00	(Referent)	1.00	(Referent)	
Yes	10,389	4.0	0.42	(0.34, 0.51)	0.43	(0.33, 0.55)	
Hand-held phone ban in 25–69 year old drivers							
No	150,269	6.3	1.00	(Referent)	1.00	(Referent)	
Yes	65,033	2.6	0.42	(0.36, 0.48)	0.39	(0.33, 0.46)	
Hand-held phone ban in >70 year old drivers							
No	12,027	1.4	1.00	(Referent)	1.00	(Referent)	
Yes	4256	0.4	0.67	(0.23, 1.93)	0.64	(0.24, 1.68)	

^a^The total number of drivers with the specified characteristic by presence/absence of hand-held cell phone use while driving legislation

^b^Percentage of drivers who were observed engaging in hand-held cell phone conversations by presence/absence of hand-held cell phone use while driving legislation

^c^All crude and adjusted odds ratios were calculated using logistic regression for complex surveys; adjusted models controlled for year, sex, race, urbanicity of location, seatbelt use, vehicle type, presence of universal texting ban (binary), presence of young driver all cell phone ban (binary), non-universal texting while driving law (binary), and the number of cell phone subscriptions per 100 residents

^d^The *p*-value presented is from the interaction term which assessed the relationship between the sub-group and hand-held CPWD ban

In regards to drivers’ sex, sub-group differences were seen (Table 3). Females talked on hand-held phones more than males irrespective of whether a hand-held ban was in existence. Although, hand-held CPWD bans were associated with lower hand-held phone conversations for both sexes. Hand-held phone conversations were particularly lower among women drivers when these bans were present.

**Table 3:** The association between driver hand-held cell phone conversations and state legislation stratified by sex

Characteristic	Total N^a^	Percent of drivers holding phone to ear^b^	Crude Odds Ratio (95% Confidence Limit)^c^		Adjusted Odds Ratio (95% Confidence Limit)^c^		*P*-value^d^
Hand-held phone ban in male drivers							<0.0001
No	106,969	4.9	1.00	(Referent)	1.00	(Referent)	
Yes	45,615	2.4	0.50	(0.43, 0.58)	0.47	(0.40, 0.55)	
Hand-held phone ban in female drivers							
No	77,026	8.0	1.00	(Referent)	1.00	(Referent)	
Yes	34,063	3.0	0.36	(0.31, 0.43)	0.34	(0.28, 0.41)	

^a^The total number of drivers with the specified characteristic by presence/absence of hand-held cell phone use while driving legislation

^b^Percentage of drivers who were observed engaging in hand-held cell phone conversations by presence/absence of hand-held cell phone use while driving legislation

^c^All crude and adjusted odds ratios were calculated using logistic regression for complex surveys; adjusted models controlled for year, age, race, urbanicity of location, seatbelt use, vehicle type, presence of universal texting ban (binary), presence of young driver all cell phone ban (binary), non-universal texting while driving law (binary), and the number of cell phone subscriptions per 100 residents

^d^The *p*-value presented is from the interaction term which assessed the relationship between the sub-group and hand-held CPWD ban

In reference to drivers’ race, no sub-group differences were noted (*p* = 0.3036). While African American drivers used cell phones more than other racial groups, these bans were associated with lower roadside observed cell phone use for all racial groups (Table 4).

**Table 4:** The association between driver hand-held cell phone conversations and state legislation stratified by race

Characteristic	Total N^a^	Percent of drivers holding phone to ear^b^	Crude Odds Ratio (95% Confidence Limit)^c^		Adjusted Odds Ratio (95% Confidence Limit)^c^		*P*-value^d^
Hand-held phone ban in White drivers							0.3036
No	153,950	6.0	1.00	(Referent)	1.00	(Referent)	
Yes	58,863	2.6	0.43	(0.37, 0.50)	0.37	(0.32, 0.45)	
Hand-held phone ban in African American drivers							
No	18,334	8.0	1.00	(Referent)	1.00	(Referent)	
Yes	5700	3.7	0.53	(0.40, 0.69)	0.62	(0.45, 0.87)	
Hand-held phone ban in Other drivers							
No	11,711	6.4	1.00	(Referent)	1.00	(Referent)	
Yes	15,115	2.4	0.38	(0.29, 0.51)	0.43	(0.30, 0.60)	

^a^The total number of drivers with the specified characteristic by presence/absence of hand-held cell phone use while driving legislation

^b^Percentage of drivers who were observed engaging in hand-held cell phone conversations by presence/absence of hand-held cell phone use while driving legislation

^c^All crude and adjusted odds ratios were calculated using logistic regression for complex surveys; adjusted models controlled for year, sex, age, urbanicity of location, seatbelt use, vehicle type, presence of universal texting ban (binary), presence of young driver all cell phone ban (binary), non-universal texting while driving law (binary), and the number of cell phone subscriptions per 100 residents

^d^The *p*-value presented is from the interaction term which assessed the relationship between the sub-group and hand-held CPWD ban

As for driver location and region, hand-held CPWD bans were equally associated with lower hand-held cell phone conversations across rural, suburban, and urban locations (*p* = 0.9290; table not shown). While hand-held cell phone bans were associated with lower driver cell phone use in all regions (Table 5), they were particularly lower in Western states (*p* = 0.0003). Drivers in Western states with hand-held bans were associated with 69% lower cell phone use while driving compared to 53% and 50% lower use in the Northeast and South, respectively.

**Table 5:** The association between driver hand-held cell phone conversations and state legislation stratified by geographic region

Characteristic	Total N^a^	Percent of drivers holding phone to ear^b^	Crude Odds Ratio (95% Confidence Limit)^c^		Adjusted Odds Ratio (95% Confidence Limit)^c^		*P*-value^d^
Hand-held phone ban in Northeast drivers							0.0003
No	35,126	5.9	1.00	(Referent)	1.00	(Referent)	
Yes	31,483	2.6	0.53	(0.39, 0.73)	0.47	(0.30, 0.72)	
Hand-held phone ban in Midwestern drivers							
No	60,353	5.2	1.00	(Referent)	1.00	(Referent)	
Yes	N/A	N/A	N/A	N/A	N/A	N/A	
Hand-held phone ban in Southern drivers							
No	62,644	7.0	1.00	(Referent)	1.00	(Referent)	
Yes	8484	3.7	0.54	(0.44, 0.64)	0.50	(0.38, 0.66)	
Hand-held phone ban in Western drivers							
No	25,872	7.0	1.00	(Referent)	1.00	(Referent)	
Yes	39,711	2.5	0.32	(0.26, 0.39)	0.31	(0.25, 0.38)	

*Abbreviations*: *N/A* not applicable; no state had a hand-held cell phone use while driving ban in this region effective during the study period

^a^The total number of drivers with the specified characteristic by presence/absence of hand-held cell phone use while driving legislation

^b^Percentage of drivers who were observed engaging in hand-held cell phone conversations by presence/absence of hand-held cell phone use while driving legislation

^c^All crude and adjusted odds ratios were calculated using logistic regression for complex surveys; adjusted models controlled for year, age, sex, race, urbanicity of location, seatbelt use, vehicle type, presence of universal texting ban (binary), presence of young driver all cell phone ban (binary), non-universal texting while driving law (binary), and the number of cell phone subscriptions per 100 residents

^d^The *p*-value presented is from the interaction term which assessed the relationship between the sub-group and hand-held CPWD ban

## Discussion

The findings of this analysis show that hand-held cell phone use while driving bans were associated with lower road-side observed hand-held cell phone conversations amongst drivers regardless of age, sex, race, location, and region. Although, when these bans were in effect, the occurrence of cell phone conversations were particularly lower amongst female drivers and drivers in Western states. While future interventional or educational efforts could be focused on all drivers, younger drivers, females, and African Americans engaged in hand-held cell phone conversations more than other groups and may benefit from directed efforts.

Other studies utilizing road-side observed data have also shown that universal hand-held CPWD bans were associated with lower occurrences of hand-held cell phone conversations amongst drivers. Studies by the Insurance Institute of Highway Safety have shown that after hand-held CPWD bans were passed in New York, the District of Columbia, and Connecticut, driver cell phone use rates immediately dropped 47%, 41%, and 76%, respectively [19–22]. The study conducted in New York showed that when the data were stratified, there was ~50% reduction in phone usage across both sexes and for drivers <25 and 25–59 years after the law was passed [19]. Another study by Cheng showed that driver cell phone use rates were 45% and 40% less, respectively, for 16–24 and 25+ year old drivers in states with hand-held CPWD bans [16]. The findings of this study showed that drivers in states with hand-held CPWD bans were observed conversing ~50% less compared to states without bans across most of the demographic groups. Additionally, other studies have also suggested that road side observed cell phone conversations were higher in females and younger drivers [17, 18, 20–22], which was also seen in this analysis.

There may be a potential explanation as to why hand-held CPWD laws were associated with lower occurrences of cell phone conversation across most drivers. As evident by the number of Americans who own cell phones and engage in its various forms of communication (i.e. texting, calling, email, video chatting, etc.), cell phones are part of the current culture [13]. Recent surveys have shown that this technology is widely accepted and frequently used across all ages, sexes, races, etc. [2, 3]. National surveys show that most drivers, irrespective of demographics, acknowledge these behaviors are a threat to their personal safety and disapprove of others engaging in this technology while driving [4]. In a 2014 national survey of drivers, nearly 70% of those surveyed supported bans on hand-held CPWD and >90% supported texting while driving legislation [4]. It is possible that this widespread use of this technology, self-awareness of the consequences, and support of legislation may have discouraged the behavior across the populous.

There are also possible explanations why cell phone conversations were particularly lower among female drivers and those from Western states when hand-held bans were effective. Several studies have investigated how gender/sex, and age relate to an individuals’ abatement of traffic safety laws; these studies have shown that females typically abide by traffic laws that are safety-driven more than males [37–39]. While females typically drive less than males across the age span and spend less time ‘at risk’, women also do typically receive traffic citations less than men, which may suggest that females are more compliant [25, 26]. Studies of high-visibility enforcement of cell phone use while driving laws have shown in certain areas that women drivers will obey the restrictions more than males [40, 41].

As for the regional differences, most of the universal hand-held cell phone use while driving bans have been instituted on the east and west the coasts of the U.S. during this study period (Fig. 1). It is possible that Western states may have lower occurrences of hand-held phone conversations after the enactment of hand-held cell phone bans because these states have similar legislation. Compared to the Northeast, a larger, more contiguous area is covered by the Western states’ hand-held bans. In the Northeast, states are smaller and many may commute between states that may have different laws and thus permit different behaviors. It is also possible that enforcement is higher in Western states, though this is unknown.

### Limitations

One of the strengths of this study is that it utilized a nationally representative sample of road-side observed drivers to assess the association between hand-held phone conversations and universal hand-held cell phone use while driving bans, which are effective in a limited number of states. This study also controlled for other cell phone use while driving bans, such as texting and young driver all cell phone bans, which may have confounded this relationship. Despite these strengths, this study has several inherent limitations. First, the road-side observations were likely imperfect and observers may have misclassified some drivers’ ages, sex, or races. However, these observers were trained and drivers were categorized into broad groups; systematic differences are unlikely between states with and without legislation. Second, while the survey was nationally representative, not all states were sampled. For example, Delaware, Hawaii, Vermont, and the District of Columbia all have hand-held bans, but were not sampled. Additionally, New Hampshire and Illinois were sampled, but had bans effective after the study period. Third, these observations occurred at controlled intersections where drivers may have been more inclined to make a call when stopped. Fourth, these observations only occurred during day light hours. Based on other traffic safety law research, it is likely that night time and day time driving behaviors differ [42, 43]. Therefore, these findings may not generalizable to nighttime observations. Fifth, this study did not control for the level of enforcement or drivers’ exposure to media/educational campaigns regarding distracted driving, as this was virtually impossible; these activities may have influenced certain drivers’ behaviors and not the laws themselves. Although, enforcement of cell phone use while driving laws appears low [44] and nearly all states spend money on distracted driving campaigns and driver education [45]. Lastly, it cannot be inferred that hand-held cell phone use while driving bans caused drivers to converse less on their cell phones. These findings are clearly associative.

## Conclusions

The findings of this analysis suggest that universal hand-held cell phone use while driving bans were associated with markedly lower hand-held cell phone conversations across all drivers, including those of different ages, sexes, races, and geographic locations. However, sub-groups differences were seen by sex and by region. As road-side observed cell phone use was higher overall among females, younger age groups, and African American drivers, these groups may benefit from directed interventional efforts.

## Appendix

**Table 6:** Effective dates and U.S. Census region of states sampled in the 2008–2013 National Occupant Protection Use Survey, United States

State	Effective dates of universal hand-held cell phone use while driving bans	U.S Census Region
Alabama	NA	South
Arkansas	NA	South
California	07/01/08	West
Colorado	NA	West
Connecticut	10/01/05	Northeast
Florida	NA	South
Georgia	NA	South
Illinois^a^	NA	Midwest
Indiana	NA	Midwest
Kentucky	NA	South
Maine	NA	Northeast
Maryland	10/01/10	South
Massachusetts	NA	Northeast
Michigan	NA	Midwest
Minnesota	NA	Midwest
Mississippi	NA	South
Missouri	NA	Midwest
North Carolina	NA	South
North Dakota	NA	Midwest
Nebraska	NA	Midwest
Nevada	01/01/12	West
New Hampshire^a^	NA	Northeast
New Jersey	07/01/04	Northeast
New Mexico	NA	West
New York	11/01/01	Northeast
Ohio	NA	Midwest
Oklahoma	NA	South
Oregon	01/01/10	West
Pennsylvania	NA	Northeast
South Carolina	NA	South
Texas	NA	South
Utah	NA	West
Washington	07/01/08	West
West Virginia	07/01/12	South
Wisconsin	NA	Midwest

*Abbreviations*: *NA* not applicable; no universal hand-held ban exists

^a^: Illinois and New Hampshire implemented hand-held cell phone bans after 2013

## Abbreviations

CPWD: Cell phone use while driving
NHTSA: National Highway Traffic Safety Administration
NOPUS: National Occupant Protection Use Survey; US = United States
